# Supplementary material for: One-Step Lignin Refining Process: The Influence of the Solvent Nature on the Properties and Quality of Fractions
Source: Polymers (Basel). 2022 Jun 11;14(12):2363. doi: 10.3390/polym14122363 (PMC9227930; doi:10.3390/polym14122363)
Supplement: Supplementary file 1 [file polymers-14-02363-s001.zip › polymers-1748530-supplementary.pdf]

Article

One-step Lignin Refining Process: the Influence of the Solvent Nature on the Properties and Quality of Fractions

Oihana Gordobil[1*], René Herrera[1,2], Jakub Sandak[1,3] and Anna Sandak[1,4]

[1] InnoRenew CoE, Livade 6, 6310, Izola, Slovenia

[2] Department of Chemical and Environmental Engineering, University of the Basque Country, Plaza Europa, 1, 20018, Donostia-San Sebastian, Spain;

[3] University of Primorska, Andrej Marušič Institute, Titov trg 4, 6000 Koper, Slovenia

[4] University of Primorska, Faculty of Mathematics, Natural Sciences, and Information Technologies, Glagoljaska 8, 6000 Koper, Slovenia

*Correspondence: Oihana.gordobil@innorenew.eu

Supporting Information

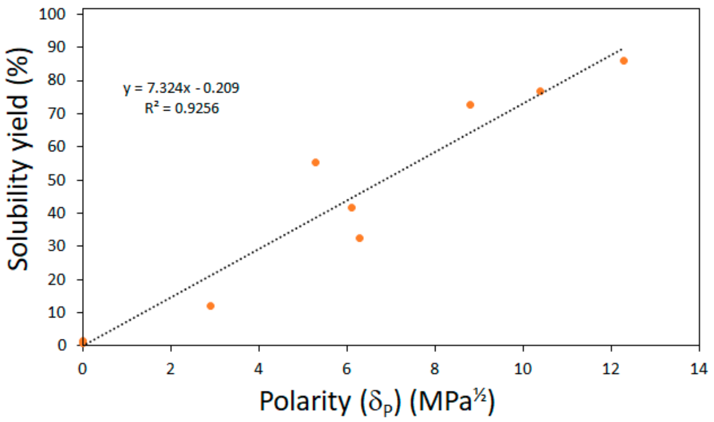

Figure S1. Correlation between the solubility yield and the polarity of the solvents.

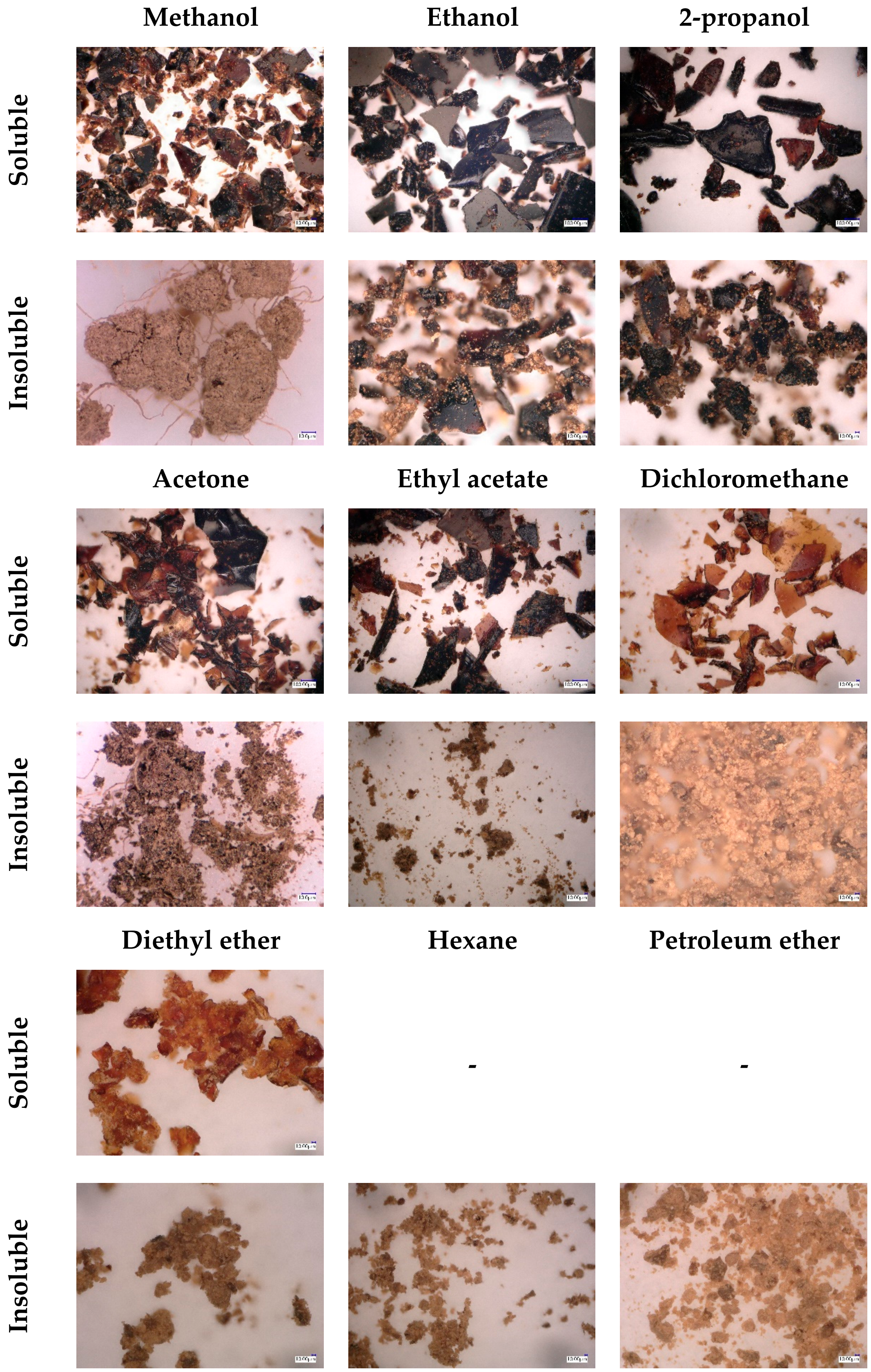

Figure S2. Micrographs of fractionated lignin samples.

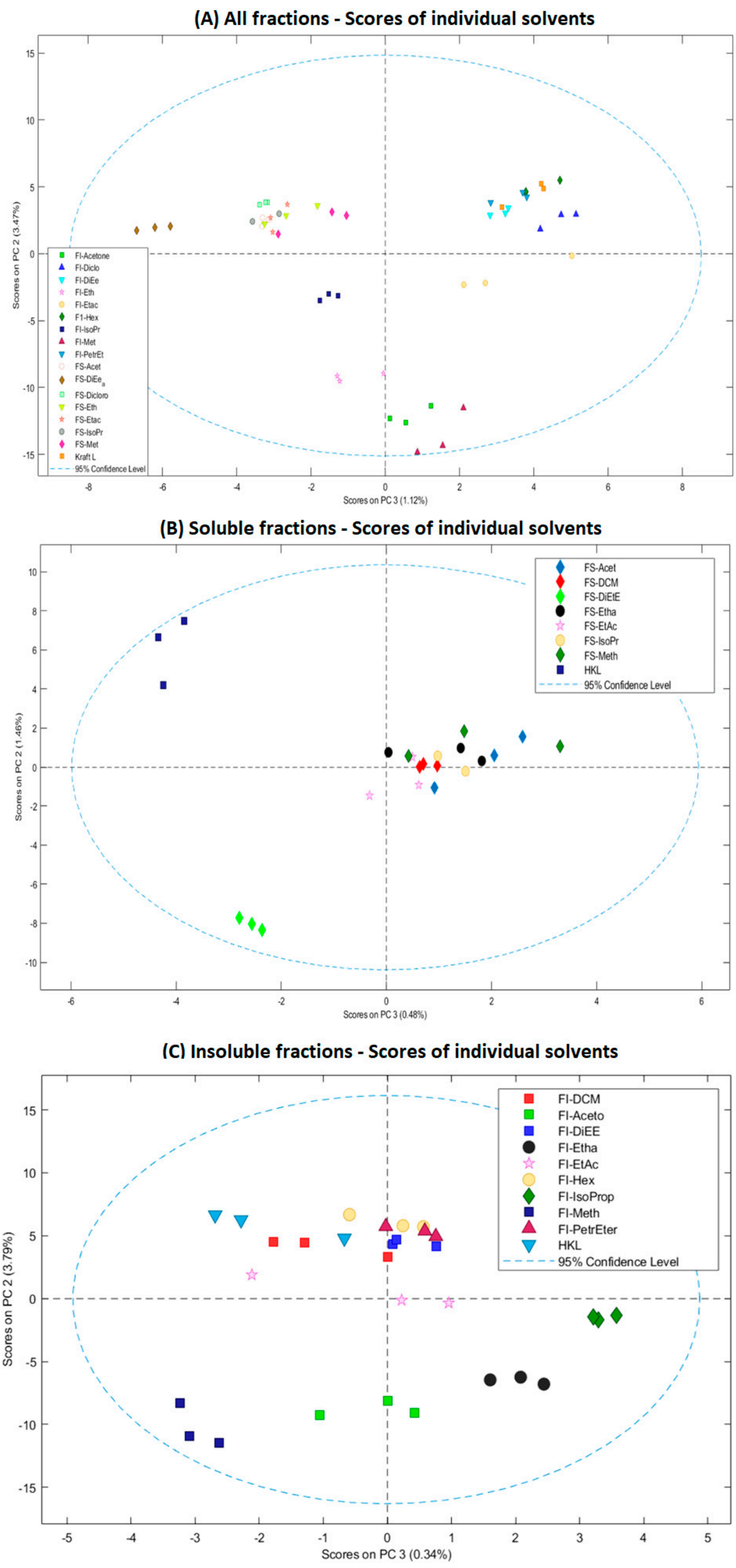

Figure S3. Scores plot of all samples classified according to the different solvents used.

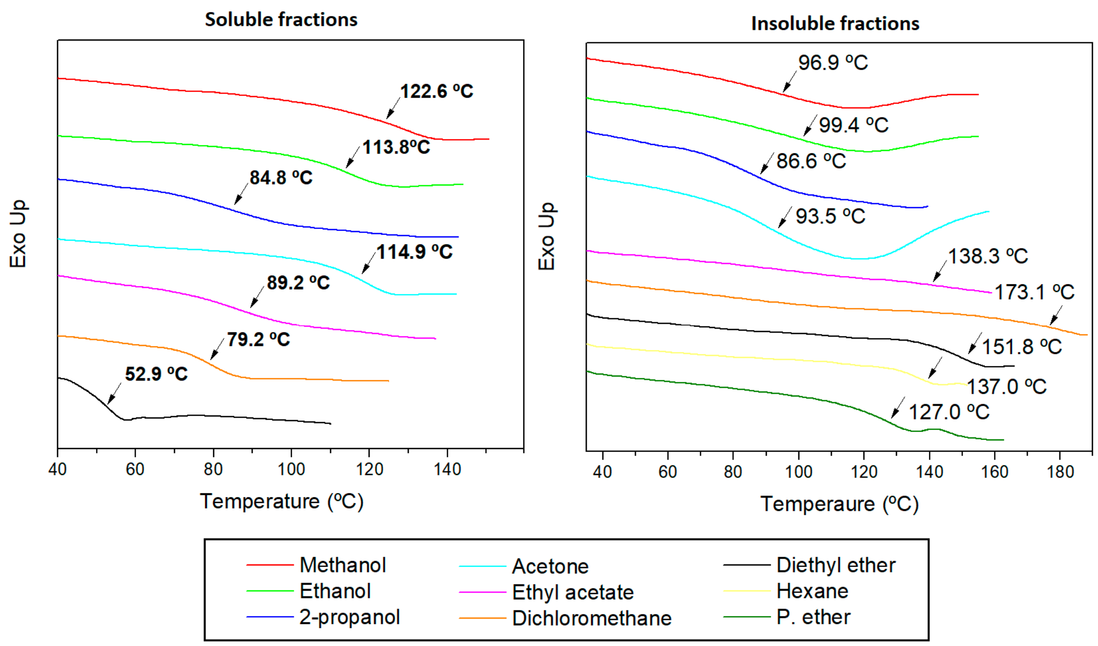

Figure S4. DSC curves of lignin fractions.

Table S1. Temperatures, char residue characteristics and ash content of crude HKL and soluble fractions.

	T_{10} (°C)	T_{50} (°C)	T_{max} (°C)	Residue at 800 °C (%)	Ash (%)
HKL	241.9 ± 1.2	403.9 ± 0.9	328.4 ± 2.2	33.1 ± 1.1	2.6± 0.3
Methanol	249.7 ± 4.8	436.8 ± 2.7	325.4 ± 3.6	34.8 ± 3.0	1.4 ± 0.2
Ethanol	250.7 ± 3.4	419.4 ± 9.7	334.8 ± 1.0	33.2 ± 4.9	0.7 ± 0.1
2-propanol	230.3 ± 3.4	363.2 ± 6.3	343.4 ± 9.4	28.2 ± 4.3	0.4 ± 0.2
Acetone	248.5 ± 3.1	414.8 ± 8.5	333.7 ± 3.4	36.2 ± 0.8	0.7 ± 0.3
Ethyl acetate	235.9 ± 7.1	377.3 ± 6.1	344.3 ± 7.0	31.4 ± 1.4	0.4 ± 0.1
Dichloromethane	249.9 ± 3.3	356.8 ± 2.9	326.0 ± 9.1	26.1 ± 3.6	0.3 ± 0.0
Diethyl ether	197.2 ± 9.4	322.7 ± 5.7	324.2 ± 7.1	13.0 ± 0.0	0.1 ± 0.0

Table S2. Temperatures, char residue characteristics and ash content of insoluble fractions.

	T_{10} (°C)	T_{50} (°C)	T_{max} (°C)	Residue at 800 °C (%)	Ash (%)
HKL	241.9 ± 1.2	403.9 ± 0.9	328.4 ± 2.2	33.1 ± 1.1	2.6± 0.3
Methanol	228.2 ± 0.0	295.6 ± 2.7	290.0 ± 5.5	13.3 ± 0.9	8.2 ± 0.4
Ethanol	239.4 ± 6.1	403.3 ± 4.9	280.4 ± 2.3	32.7 ± 1.2	6.6 ± 0.1
2-propanol	243.3 ± 2.2	458.2 ± 9.9	307.2 ± 0.8	39.6 ± 0.3	3.4 ± 0.1
Acetone	221.1 ± 7.2	337.0 ± 5.2	302.4 ± 1.1	26.3 ± 0.5	7.9 ± 0.2
Ethyl acetate	232.0 ± 2.3	437.8 ± 9.8	290.3 ± 2.4	36.6 ± 1.2	4.0 ± 0.2
Dichloromethane	250.5 ± 2.5	458.2 ± 3.4	279.5 ± 0.7	38.8 ± 3.0	3.3 ± 0.5
Diethyl ether	251.7 ± 1.3	432.3 ± 7.9	329.0 ± 3.2	38.4 ± 0.9	2.6 ± 0.1
Hexane	241.8 ± 1.1	431.0 ± 0.1	331.4 ± 3.8	38.0 ± 0.6	2.7 ± 0.9
P. ether	237.8 ± 7.2	418.7 ± 5.2	331.4 ± 1.7	31.5 ± 3.2	2.2 ± 0.1
